# Polymer-Nanoparticle Composites: From Synthesis to Modern Applications

**DOI:** 10.3390/ma3063468

**Published:** 2010-05-28

**Authors:** Thomas Hanemann, Dorothée Vinga Szabó

**Affiliations:** 1 Institute for Materials Research, Karlsruhe Institute of Technology (KIT), Hermann-von-Helmholtz-Platz 1, D-76344 Eggenstein-Leopoldshafen, Germany; E-Mail: dorothee.szabo@kit.edu (D.V.S.); 2 Institute for Microsystems Engineering (IMTEK), University of Freiburg, Georges-Koehler-Allee 102, D-79110 Freiburg, Germany

**Keywords:** nanocomposites, polymer matrix, spherical nanoparticles, physical property tailoring, polymer-nanoparticle-interface

## Abstract

The addition of inorganic spherical nanoparticles to polymers allows the modification of the polymers physical properties as well as the implementation of new features in the polymer matrix. This review article covers considerations on special features of inorganic nanoparticles, the most important synthesis methods for ceramic nanoparticles and nanocomposites, nanoparticle surface modification, and composite formation, including drawbacks. Classical nanocomposite properties, as thermomechanical, dielectric, conductive, magnetic, as well as optical properties, will be summarized. Finally, typical existing and potential applications will be shown with the focus on new and innovative applications, like in energy storage systems.

## 1. Introduction

Within the last 15 years, materials and structures showing geometric dimensions below 100 nm have gained more and more attraction to the scientific world and stimulated spirit of research on sometimes fancy ideas for future applications like molecular manufacturing or space elevators as well as on serious products for consumer goods, health, medical or food technology [1,2,3,4,5]. With respect to the almost infinite numbers of scientific reports, books, and journal contributions on nanoscience and nanotechnology, the authors of this review article concentrate on some elementary considerations on inorganic nanoparticle properties, basic remarks on synthesis and processing challenges, functional properties and applications of polymer-nanoparticle-composites, as well as on modern research fields, where these polymer matrix composites play a decisive role:

- optical and magnetic properties
- microelectronic devices
- piezoelectric actuators and sensors
- electrolytes, anodes in lithium-ion-batteries and supercapacitors
- organic solar cells and intrinsic conductive polymers
- photoresists used in microelectronics and microsystems technologies
- biomedical sciences.

Before discussing various synthesis methods and properties of nanocomposites, one has to consider elementary consequences of the small size of nanoparticles. Nanoparticles are, by definition, particles with diameters below the micron dimension: generally, below 0.1 µm (100 nm). A more stringent definition considers nanoparticles as particles with properties depending directly on their size. Examples are optical, electrical, or magnetic properties. Therefore, in many cases the latter definition restricts nanoparticles to particles with sizes below 10–20 nm. Additionally, with decreasing particle size, the ratio of surface/volume increases, so that surface properties become crucial. The dependency of surface/volume ratio is a function of size. In this context, it is important to realize that e.g., 5 nm particles consist of only a few 1000 atoms or unit cells and possess approximately 40% of their atoms at the surface. In contrast, 0.1 µm particles contain some 10^7^ atoms or unit cells, and only 1% of their atoms are located at the surface. Therefore, the smaller the particles are, the more important will be surface properties, influencing interfacial properties, agglomeration behavior, and also - as will be shown later - physical properties of the particles. As the surface area of nanoparticles is some 100 m^2^/g, contaminations stemming from the various synthesis processes, as e.g., remaining precursor residuals, or solvents, may additionally influence the surface properties. 

**Figure 1:**
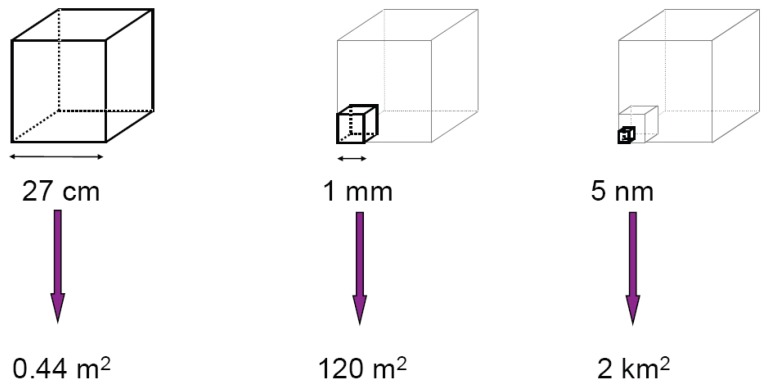
Schematic representation of the increasing surface area while decreasing particle size, using a 50 kg quartz cube. The cubes are not true to scale.

A very demonstrative example of the influence of surface area, adapted from [6], is to visualize a 50 kg piece of quartz (SiO_2_) in the form of a cube. This cube has a total edge length of about 27 cm. As a single crystal, this piece of quartz would have a total surface area of about 0.44 m^2^. Reducing the edge length of the contributing cubes (corresponding to crystal size) to 1 mm, the quartz cube would consist of approximately 2 × 10^7^ small cubes with a total surface area of approximately 120 m^2^. A further reduction to 5 nm would lead to approximately 1.6 × 10^23^ very small cubes with a total surface area of around 2 km^2^. This is shown schematically in Figure 1.

Such extremely small particles possess only poor “compacting properties”. The powder density is very low, so that 100 mg of a nanopowder may take a volume of around 1 cm^3^. In the ideal case, assuming monomodal spherical nanoparticles, no friction between the particles, no van-der-Waals forces between the particles, no agglomeration, and a cubic face centered arrangement of particles; a maximum filling degree of 74 vol % can be obtained for a composite. In reality, the filling degree will always be significantly lower.

In addition to the established main material classes of metals, ceramics and polymers, composites, especially polymer-matrix composites (PMC), allow for a physical property tailoring using different type of fillers [7,8]. Depending on the particle size, particle shape, specific surface area and chemical nature, the following polymer matrix properties can be modified:

- electrical and thermal conductivity
- polymer phase behavior and thermal stability
- mechanical properties like stiffness, Young’s modulus, wear, fatigue, and others
- flame retardancy [9]
- density
- physical properties such as magnetic, optic, or dielectric properties.

In principle, the whole bandwidth of polymer processing technology can be used for shaping, molding or replication of the polymer-based composites enabling a low cost fabrication of components and devices. On the one hand new potential applications can be realized using nanoparticles with small sizes, but on the other hand they complicate the realization of homogeneous and highly filled composites. Comprehensive books and reviews covering polymer matrix composites containing different kinds of nanosized fillers like clay, carbon nanotubes, and others, can be found in [9,10,11].

Depending on the synthesis conditions and the surface chemistry, the nanoparticles tend to form soft or hard agglomerates. Hard agglomerates consist of smaller particles which are connected to each other by sinter necks. They can be destroyed only by high energy milling. Soft agglomerates are accumulations of isolated particles which are connected to each other by attractive physical interactions like van-der-Waals or hydrogen bridge forces. Soft agglomerates can be disrupted into smaller particles by shear forces generating mechanical stress gradients. The interparticle interactions depend mainly on the particles surface chemistry, the shape, aspect ratio and dimensionality, the interparticle distance and the polydispersity [12].

## 2. Special Features of Nanoparticles

### 2.1. Particle size dependent properties of inorganic nanoparticles

Ensembles of isolated nanoparticles with particle sizes below around 20 nm exhibit physical properties that may differ from their bulk counterparts. The effects are sometimes crucial, as they will strongly influence the desired or expected property of the nanocomposite. A significant influence of particle size is observed as well as on magnetic, dielectric, electronic, optical, thermodynamic, and thermomechanical, and on structural properties. The following explanations rely on general features, found in metallic, ceramic and semiconducting nanoparticles.

**Figure 2:**
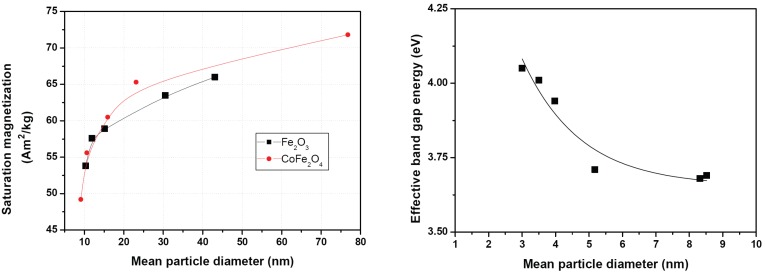
Examples for typical particle size-dependent physical properties. Left: Saturation magnetization as a function of particle size. Data taken from [15]. Right: Band gap energy for SnO_2_ as a function of particle size. Data taken from [19].

Size-dependent magnetic properties have been studied for around two decades. Tang *et al.* [13,14] reported an increasing saturation magnetization in the particle size range from 7.5 nm to 25 nm. In this size regime, the authors also observed a decrease of the transition temperature. Han *et al.* described similar behavior for Co-containing ferrite nanoparticles [15]. The size dependence of saturation magnetization is depicted exemplarily in Figure 2 (left). These dependencies can be stated as general rules as nanoparticles are typically covered by a 0.5 to 1 nm thin, nonmagnetic surface layer. As the amount of surface increases with decreasing particle size, the ratio of nonmagnetic surface layer to magnetic material also increases.

Size-dependent refractive indices were reported for narrow band-gap semiconducting nanoparticles such as PbS by Kyprianidou-Leodidou *et al.* [16]. Above 25 nm particle size the refractive index of PbS at different wavelengths was more or less independent of the particle size, and near the bulk values, respectively. For PbS particles with diameters below 25 nm the refractive indices decreased significantly with size. Similar observations were made from these authors featuring the absorption coefficient. In Si-nanoclusters a significant luminescence peak blue-shift was calculated for decreasing particle size. In parallel, the spectra became broader with decreasing particle size. These effects were described in the size regime from 2 to 6 nm [17]. Theoretical considerations predicted size-dependent energy band gap and dielectric constants for semiconducting nanoparticles [18]. Lee *et al.* [19] studied the size dependence of band gap energies in SnO_2_ quantum dots. Figure 2 (right) shows the significant increasing band gap energy with decreasing particle size. Nienhaus *et al.* [20] and Szabó *et al.* [21] observed a blue shift of the plasmon losses with decreasing particle size in SnO_2_.

Concerning thermodynamic properties such as phase transitions or phase stabilities, interesting observations were made for materials existing in several polymorphs. The physical properties such as optoelectronic, photochemical or catalytic properties may be influenced by phase as well as by size. This is the case for ZrO_2_ and TiO_2_, both existing in different phases, and very interesting as nanofillers in composites. Suresh *et al.* [22] described an inverse relationship between transformation temperature and particle size in ZrO_2_, and deduced a grain size dependent phase diagram. Li *et al.* [23] made energetic considerations and calculated decreasing transition temperatures with decreasing particle sizes for nanoscaled ZrO_2_. Zhang and Banfield [24] analyzed the phase stability of nanocrystalline TiO_2_. They found anatase to be more stable than rutile when the particle size decreased below around 14 nm. Phase stabilities of TiO_2_ and ZrO_2_ were also investigated by Schlabach *et al.* [25,26]. Both ceramics were found to occur in non-typical phases as nanoparticles compared to the bulk material and are subject to phase transformation and grain growth with increasing temperature. Coating the nanoparticles with a different ceramic layer suppresses phase transformations and obstructs grain growth.

The knowledge about which phase is stable under which conditions is in-so-far important, as TiO_2_ is frequently used as filler to modify optical properties of polymers. The phases differ in their refractive indices: bulk anatase is characterized by a refractive index of 2.54 (at 550 nm) and a band gap of 3.20 eV, whereas rutile is characterized by a refractive index of 2.75 (at 550 nm) and a band gap of 3.03 eV for bulk, respectively. For amorphous thin TiO_2_ films a refractive index of 2.51(at 550 nm) and a band gap of 3.27 eV were reported [27].

Size effects regarding electrochemical properties and cycling stability were described for nanoscaled TiO_2_ [28,29]. With decreasing anatase particle size from 30 m to 6 nm, an increase of capacity was observed, indicating an improved lithium storage capability [28]. Similar effects were observed for rutile [29]. Here the authors found a significant increase in capacity with decreasing size from 300 nm to 15 nm for rutile particles. As both phases were cycled under different conditions, the results cannot be compared directly. Deng *et al.* [30] comment that anatase - among all different TiO_2_ phases - presents the most interesting potential regarding electrochemical properties.

Size effects for dielectric properties were found in a different size range. Chattopadhyay *et al.* [31] observed a decreasing ferroelectric to paraelectric phase transition in PbZrO_3_ at particles sizes below 100 nm, and a decrease in dielectric constant. These observations were in parallel with a decreasing pseudo-tetragonal distortion of the crystal lattice. Yan *et al.* observed a particle size dependent existence of phases in BaTiO_3_ [32]. Below a particle size of 70 nm the paraelectric phase was stable; above 100 nm the tetragonal ferroelectric phase was stable. Wada *et al.* reported about a maximum dielectric constant in BaTiO_3_ occurring at particle sizes of 70 nm or 140 nm, depending on particle synthesis method [33].

### 2.2. Polymer-nanoparticle interface

In the last few years, many outstanding and comprehensive reviews dealing with polymer-nanoparticle composites had been published, e.g., by Caseri [34]. The large specific surface area of the filler causes the formation of an interfacial polymer layer (shell) attached to the particle core [35]. Consequently, one should speak about core-shell particles dispersed in a polymer matrix. The presence of this shell also will reduce the maximum filling degree of nanoparticles in a polymer matrix. The physical properties of the polymer localized in the shell are different from the bulk polymer due to immobilization. If there are attractive forces between the filler and the interfacial polymer, the mobility of the polymer chains is reduced and the glass transition temperature increases. If there are repulsive forces between the particle and the interfacial layer, the polymer chain mobility is increased yielding in a plasticizing effect with glass transition temperature depression. Especially precise differential scanning calorimetry (DSC) and dynamic mechanical analysis (DMA) measurements can be used for a measurement of the glass transition temperature change with nanofiller load [35]. There is strong evidence that the interaction of the interfacial layer with the particle and the free bulk polymer is responsible for the changes in thermomechanical and electrical properties. Reminding the increasing specific surface area with decreasing particle size, the amount of interfacial polymer layer strongly depends on nanofiller size and load. Assuming an interfacial polymer layer thickness of 0.5 nm, a cubic faced centered arrangement of the nanoparticles, and a particle size of 50 nm, a maximum filling degree of 69.5 vol % can be reached. If the particle size decreases to 3 nm with 0.5 nm interfacial polymer layer, the maximum filling degree drops down to 31 vol %.

To adjust polymer-nanoparticle-composite properties and their processability tailoring of nanoparticle surfaces as well as tuning of the interfacial layer is crucial [34,35,36]. Also, depending on particle size, a maximum filling degree is given. 

## 3. Composite Types

In this chapter we briefly describe the main different types of nanocomposites which are discussed in this review, and will play a main role concerning property modification of polymers and applications.

### 3.1. Polymer-matrix composites

**Figure 3:**
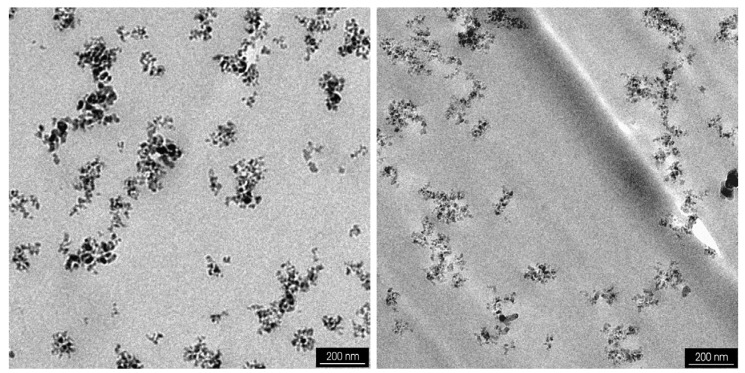
Transmission electron microscopy (TEM) micrographs of Aerosil^®^ R8200, dispersed in a methylmethacrylate (MMA) using a high speed stirrer (left) or a high pressure homogenizer (right), after solidification to polymethylmethacrylate (PMMA).

This is the classical type of a nanocomposite, where - in the ideal case - isolated nanoparticles are finely dispersed in a polymer. In reality, agglomerated nanoparticles are dispersed in a polymer matrix. The degree of agglomeration can be influenced, as will be shown in section 4.4. Figure 3 shows an example of such a polymer-matrix nanocomposite using commercial nanoparticles. Functional nanocomposites with improved physical properties allow new applications e.g., in microoptics, electronics, energy conversion or storage. In most of the cases, the change of the aspired feature correlates with the filler load. The resulting composite flow behavior limits mostly huge solid loadings and therefore property adjustment due to restrictions in shaping or molding. Shear rate- and temperature-dependent as well as oscillatory rheological investigations are therefore necessary for a detailed description of the composites flow properties prior to shape forming [37,38]. In case of nanosized fillers, the specific surface area and the resulting huge polymer-filler interfacial layer dominates the rheological behavior.

### 3.2. Composite nanoparticles

Composite nanoparticles as core/shell nanoparticles or surface modified nanoparticles may be considered as a special type of nanocomposites. When these nanoparticles contain an inorganic core and an organic shell one may speak about hybrid nanoparticles. In this case the inorganic core may be a metal or a metal oxide, and the organic shell either a polymerized monomer, a chromophore, a detergent or surfactant, carbon, or some organic molecule. Particles of this type, consisting of a metal-oxide core and a polymerizable organic shell, were reported in the mid and late-90s by Vollath [39,40,41], synthesized in a microwave plasma reactor by gas phase synthesis. This concept allows the design of new functional materials with novel or modified magnetic, optical, electronic or biological properties. 

**Figure 4:**
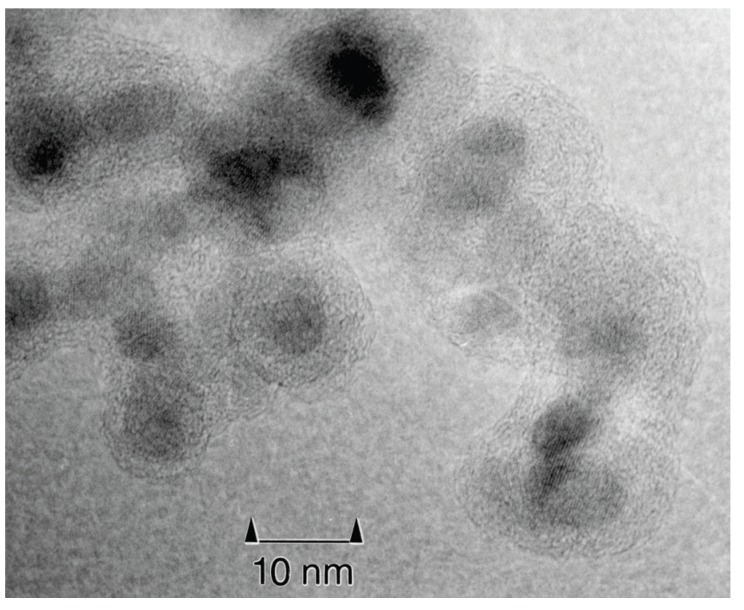
TEM micrograph of γ-Fe_2_O_3_/polymer core/shell nanoparticles.

Figure 4 visualizes exemplarily inorganic/organic core/shell nanoparticles by a transmission electron micrograph of polymer-coated γ-Fe_2_O_3_ nanoparticles. In this case, the core is around 8 nm in diameter; the polymer coating is around 2 nm in thickness.

**Table 1:** Portfolio of various core/shell hybrid nanoparticles with a ceramic core and an organic shell.

Core	Shell	Synthesis Method	Ref.
Metal-oxides	Polymerizable	Microwave Plasma plus *in situ* coating	[39]
HfO_2_, ZrO_2_, ZnO, Fe_2_O_3_, TiO_2_, Al_2_O_3_	MMA; Fluoropolymers	Microwave Plasma plus *in situ* coating	[52]
Fe_2_O_3_	Modified PMMA	Microwave Plasma plus *in situ* coating	[40]
Fe_2_O_3_	Initiator plus styrene	Complex	[55]
Al_2_O_3_	Polyacrylic acid (PAA)	Commercial nanoparticles, layer by layer deposition with controlled polymer adsorption	[42]
Al_2_O_3_	Polyethylene (PE)	*In situ* Chemical Vapor Synthesis	[50]
Al_2_O_3_	Pyrrole	*Ex situ* deposition using plasma polymerization	[49]
ZrO_2_	PE	*Ex situ* by inductively coupled plasma polymerization	[51]
TiO_2_	PMMA	*Ex situ* deposition on commercial, nanoparticles by mixing with MMA solution and irradiation with electron beam	[47]
TiO_2_	PMMA	*Ex situ* by plasma polymerization	[46]
TiO_2_	Polystyrene (PS)	*Ex situ* by radical polymerization	[44]
SiO_2_	PS	SiO_2_ by Stöber synthesis; surface modification with coupling reagent; polymerization	[43]
SiO_2_	Acrylate based polymers	*In situ* Chemical Vapor Synthesis	[48]
ZnO	Acrylic acid	*Ex situ* deposition using plasma polymerization	[45]
Fe_3_O_4_	ε-Caprolactone	Fe_3_O_4_ by alkaline hydrolysis, followed by surface functionalization with ultrasound; surface initiated ring opening polymerization	[53]
Fe_3_O_4_	ε-Caprolactone	Fe_3_O_4_ by alkaline hydrolysis, followed by surface functionalization; graft polymerization using microwaves	[54]

In the last decade, a broad portfolio of nanocomposite particles with different functional properties have been developed as shown in Table 1, depending on the inorganic core and the organic shell. Many research groups worldwide are involved in this field. Chen and Somasundaran, for example.described the preparation of Al_2_O_3_/PAA core/shell nanocomposites by a controlled polymer bridging, using commercial Al_2_O_3_ nanoparticles [42]. A polystyrene-based coating was used for SiO_2_ nanoparticles [43] and TiO_2_ nanoparticles [44], respectively. Acrylate-based nanoparticle composites were reported from ZnO [45], TiO_2_ [46,47] and SiO_2_ [48]. Al_2_O_3_ nanoparticles were coated with pyrrole [49], or *in situ* with polyethylene [50]. He *et al.* reported about ZrO_2_ nanoparticles, coated with quasi-polyethylene [51]. Various oxide nanoparticles were coated with acrylic based monomers and with fluoropolymers [52]. Schmidt developed magnetic core/shell nanoparticles based on Fe_3_O_4_ and ε-caprolactone [53] by surface initiated ring-opening polymerization, whereas Nan *et al.* [54] synthesized a similar type of nanocomposite using microwave assisted graft polymerization. Gravano *et al.* described the surface functionalization of Fe_2_O_3_ with ligands and polymers [55].

Recently, core/shell nanoparticles also became of interest for the application of anode-materials in lithium-ion-batteries. Mainly carbon as graphite, amorphous carbon, or graphene is used as the organic compound (Table 2). Fu *et al.* developed TiO_2_/C nanocomposites [56], Chen *et al.* [57] describe nanocomposites made of micron-sized graphite core and a shell of SnO_2_-nanoparticles. SnO_2_/C core/shell nanoparticles are described by Park *et al.* [58] and Qiao *et al.* [59]. Details concerning electrochemical properties will be given in section 6.5.3.

**Table 2:** Core/shell hybrid nanoparticles for application as anode material in lithium-ion-batteries.

Core	Shell	Synthesis Method	Ref.
TiO_2_	C	Emulsion polymerization plus heat treatment	[56]
C (micro-sized)	SnO_2_	Sol-gel, using commercial graphite	[57]
SnO_2_	C	Thermal evaporation	[58]
SnO_2_	C	One-pot solvothermal synthesis and subsequent calcination	[59]

### 3.3. Microsphere composite nanoparticles

**Figure 5:**
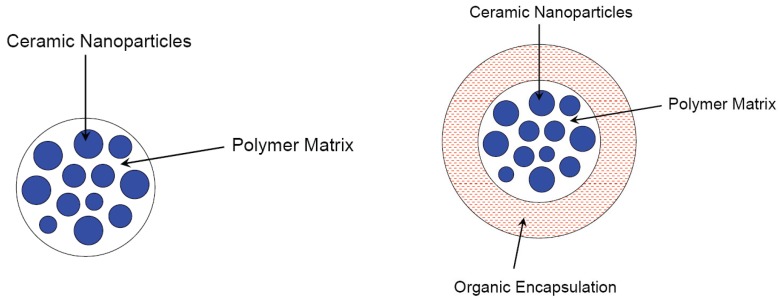
Morphologies of microsphere composite nanoparticles, schematically.

This special type of composite is characterized by larger spheres, themselves consisting of a nanocomposite. Figure 5 shows schematically the morphology of this type of composite. Microsphere composites are reported from several authors, but significantly less than core/shell nanoparticles. The ceramic part in most described cases is magnetic (either Fe_2_O_3_, or Fe_3_O_4_) with an application focus in biology or magnetic resonance. Mangeney *et al.* reported about the bioreactivity of magnetic Fe_2_O_3_-PS/polypyrrole (PPy) core/shell particles [60]. Ho and Li [61] described magnetic core/shell particles consisting of hydrophobic PMMA cores with γ-Fe_2_O_3_ nanoparticles inside. The PMMA cores were encapsulated with hydrophilic chitosan shells.

Hsieh *et al.* apply a polyaniline encapsulation for SiO_2_/γ-Fe_2_O_3_ nanoparticles [62]. Magnetic encapsulated polymer nanocomposites are prepared by Jeon *et al.* [63]. Hollow polyaniline/Fe_3_O_4_ microspheres are reported by Yang *et al.* [64]. Another approach was presented by Zhang *et al.* [65]. This research group developed Sn-nanoparticles encapsulated in hollow carbon-spheres for application in lithium-ion-batteries.

## 4. Composite Formation Techniques

In this section, the most important synthesis methods for nanocomposite formation will be briefly described. It mainly will be distinguished between *ex situ* methods, chemical *in situ* methods, and physical *in situ* methods (gas-phase methods) leading to polymer-matrix nanocomposites or nanocomposite particles. The common feature of the latter two synthesis strategies is that both start from atomic or molecular precursors to create larger building blocks. Finally, drawbacks for composite formation will be discussed.

### 4.1. *Ex situ* processes

**Figure 6:**
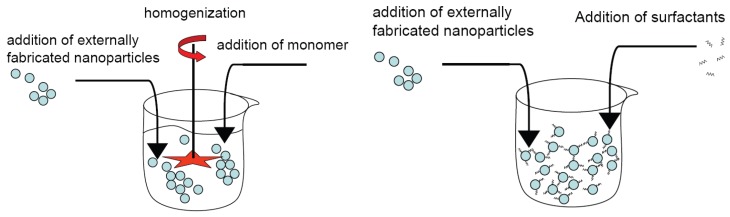
Sketch of dispersing nanoparticles in a monomer, polymer or resin (organic solution) by use of external shear forces, e.g., by a stirrer (left) or by sonification prior to polymerization. In an advanced set-up, the nanoparticles may be coated with a coupling agent/surfactant before mixing (right) with the monomer/polymer/resin.

*Ex situ* processes are generally spoken methods, where nanoparticles, synthesized in an external synthesis step, are added or mixed to a monomer or resin (organic solution), usually followed by a polymerization. This is shown schematically in Figure 6. In the simplest case the nanoparticles are used as produced or delivered, posing the most problems concerning agglomeration. Such an approach was used by Musikhin *et al.* [66] to generate luminescent polymer-dielectric nanocrystal composites using commercial Al_2_O_3_, Y_2_O_3_, ZnO and SnO_2_/Sb_2_O_3_/Sb_2_O_5_ nanoparticles, respectively. In more elaborated setups the nanoparticles were first surface functionalized and then added to the organic solution [67]. This principal method is also used by Tang and Dong [68] for the synthesis of styrene polymer/ZnO nanocomposite latex. Mahdavian *et al.* [69] encapsulate commercial Al_2_O_3_ nanoparticles by coeval use of an emulsifier with styrene/MMA using sonification and subsequent miniemulsion polymerization. Nanocomposites, consisting of epoxy thermosets and Al_2_O_3_, have been prepared by simple mixing at elevated temperatures [70,71].

Cannillo *et al.* [72] attached spherical SiO_2_ nanoparticles (100–200 nm) chemically to poly-caprolactone via grafting with a solid load of 1.0 and 2.5 wt %. Rong *et al.* [44] applied a surface functionalization to commercial TiO_2_, and then performed a free radical polymerization of styrene to generate a nanocomposite. Alternative approaches used commercial nanoparticles, applied coupling agents and finally blended the particles with polymer powder [73]. These methods lead to “bulk” composite materials. Wang *et al.* [47] combined the mixing of commercial nanoparticles in a monomer with the polymerization using electron irradiation to obtain polymer/TiO_2_ and Al_2_O_3_ nanoparticle composites. Another *ex situ* method is the coating of nanoparticles with a polymer by a subsequent polymerization treatment. An example is the coating of commercial Al_2_O_3_ nanoparticles with PAA [42] by controlled polymer bridging. Very frequently, plasma polymerization processes are used to generate core/shell type nanoparticles as shown in Table 1. Here also, externally produced nanoparticles are used. Shi *et al.* [45,49] combined a fluidized bed reactor with the classical plasma polymerization to generate polymer coated ZnO and Al_2_O_3_, respectively. He *et al.* [51] deposited a thin polymer film on ZrO_2_ nanoparticles by inductively coupled C_2_H_2_/N_2_ plasma.

### 4.2. Chemical *in situ* methods

This approach uses chemical reactions in a liquid environment to generate nanocomposites. The result may be either nanocomposite particles, or compact nanocomposite material. Very comprehensive reviews on the variety of chemical synthesis methods are given by Caseri [34] and Althues *et al.* [74].

Already in the early 1990ies Ziolo *et al.* [75,76] elaborated a one-step chemical method to synthesize fine dispersed Fe_2_O_3_ nanoparticles in a cross-linked polystyrene resin. They used a synthetic ion-exchange resin and aqueous solutions of Fe(II) or Fe(III)-chloride, respectively, to exchange the ions. Cao synthesized Fe_3_O_4_/PMMA composite particles by a one-pot hydrothermal method [77]. Guan *et al.* [78] report about the synthesis of transparent polymer nanocomposites containing ZnS using a one-pot route via *in situ* bulk polymerization.

The common feature of most materials described below, in contrast to the one described before, is, that (functionalized) nanoparticles are synthesized in a first step, mostly as a sol or dispersed in a solution, followed by a second step where a monomer or resin is added and brought to polymerization. 

Gonsalves *et al.* [79] synthesized AlN nanoparticles with a sol-gel method, and then applied an effective solution mixing method to generate a homogeneous dispersion of AlN nanoparticles in polyimide. GaN/polymer nanocomposites were synthesized by *in situ* thermal decomposition of a precursor incorporated into a copolymer [80]. Gangopadhyay and Amitabha [81] prepared colloidal solutions of Fe_2_O_3_ nanoparticles, which then were added to the conducting polymer PPy. The whole mixture finally was polymerized to obtain a nanocomposite. Xiong *et al.* [82] prepared TiO_2_/polymer nanocomposites by mixing (3-methacryloxypropyl)trimethoxysilane (MPMS)-capped acrylic resins with sol-gel synthesized TiO_2_. Quantum dot/polymer nanocomposites were synthesized by polymerization in microemulsion after synthesis of the nanoparticles by thermal decomposition of a precursor [83,84]. Althues *et al.* [85] applied a two-step process to synthesize ZnO in a colloidal suspension, which finally was photopolymerized. Jiang [86] prepared magnetic nanocomposites containing Ni_0.5_Zn_0.5_Fe_2_O_4_ nanoparticles via a wet-chemical method leading to a colloidal suspension, followed by *in situ* polymerization of a monomer. A similar method is applied by Cheng *et al.* [87] for the synthesis of ZnS containing nanocomposites. The *in situ* generation of SiO_2_ nanoparticles via sol-gel techniques in an organic solvent, which contains dissolved PMMA, lead to PMMA-nanosilica-composites after solvent evaporation and drying [88].

Chemical routes based on sol-gel processes and subsequent *in situ* polymerization are commonly used for the synthesis of hybrid nanocomposite particles and nanocomposites.

### 4.3. Physical *in situ* methods

Physical methods are mainly gas-phase methods. They are able to synthesize *in situ* functionalized or encapsulated nanoparticles, appearing as hybrid core/shell nanoparticles. Their common feature is that they apply energy to transform chemical compounds (precursor and gas) into inorganic nanoparticles, and by a subsequent coating step organic compounds are grafted on the nanoparticle surfaces for coating, encapsulation or surface functionalization.

**Figure 7:**
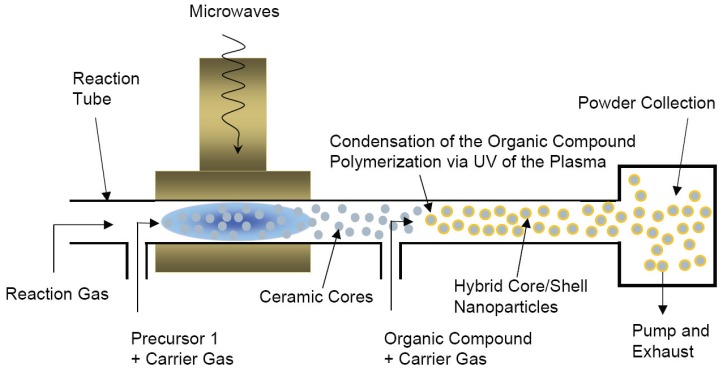
Set-up scheme for the microwave plasma synthesis of hybrid core/shell nanoparticles.

A versatile approach for the gas-phase synthesis of hybrid core/shell nanoparticles is the application of microwaves for plasma generation. This approach was developed by Vollath *et al.* [39,40,41]. The basic element of this approach is a reaction tube made of quartz glass crossing a microwave cavity. At this intersection, plasma is ignited. Volatile and water-free precursors (e.g., chlorides, carbonyls, metal-alkoxides, or metal-alkyls) are evaporated outside the reaction tube and mixed with an inert carrier gas. The components are introduced as gases into the system just in front of the plasma zone. Here. the chemical reaction in the gas-phase and the nucleation and growth of nanoparticles occurs. By using consecutive reaction zones, core/shell nanoparticles and multi-layer nanoparticles can be produced in consecutive synthesis steps. The inorganic cores are formed by homogeneous nucleation, the organic shell of hybrid nanoparticles condenses via heterogeneous nucleation and polymerizes outside of the plasma zone on the cores synthesized in the plasma (Figure 7). This approach was also used to synthesize double-coated, multifunctional core/shell nanoparticles [89,90].

Schallehn *et al.* [50] and Suffner [48] applied chemical vapor synthesis (CVS) for *in situ* polymer coating of Al_2_O_3_ and SiO_2_ nanoparticles. Instead of microwave plasma a traditional hot-wall reactor was used for inorganic nanoparticle synthesis, the coating was performed in a subsequent RF-plasma reactor. Similar setups were not only used for the synthesis of ceramic/polymer core/shell nanoparticles, but also for the production of metal/polymer core/shell nanoparticles. Srikanth *et al.* [91] used a one step microwave plasma process to encapsulate Fe nanoparticles with polystyrene. The precursor, Fe(CO)_5_, and the styrene monomer were added coevally; both components commonly passed the plasma. This process claims to be on an industrial level. Qin and Coulombe [92] applied a dual-plasma process for the synthesis of metal/organic core/shell nanoparticles. The metal (Cu) nanoparticles were synthesized through arc evaporation and vapor condensation, the subsequent organic coating was deposited by in-flight deposition of an organic compound through plasma polymerization.

### 4.4. Drawbacks in composite formation

In case of functional polymer based composites, the degree of tailored property adjustment follows mainly the volume amount of dispersed filler introducing the aspired physical property like refractive index or electrical conductivity change. Interestingly, in literature most filler amounts are not given in vol %, but in wt %. As part of a MRS Bulletin issued in 2007 Winey and Vaia collected the use of selected micro and nanosized fillers like carbon fibers and nanotubes, alumosilicates, clay as well as Al_2_O_3_ and TiO_2_ for commercial applications [93]. In the following the impact of spherical nanoparticles on the composite flow behavior, which determines the maximum accessible solid load and shaping process significantly, will be discussed.

Many *ex situ* processes generally suffer extremely from the high agglomeration tendency of nanoparticles, as it is rather difficult to destroy the nanoparticle agglomerates even using high external shear forces. In case of chemically identical materials, the interaction energy between two particles increases significantly from zero dimensional spherical particles to two-dimensional nanoscaled sheets and therefore the required dispersion effort in a polymer matrix is raised, also. Considering solvent-free composites the composite viscosity depends on the used polymer matrix (curable low viscous reactive resins or polymer melts) and the suspended filler. The expected composite viscosity determines the applied dispersing technology. Low viscous reactive resin based mixtures can be processed with dissolver stirrers or by sonification under ambient or slight elevated temperatures avoiding monomer evaporation. Different dispersing techniques with increasing shear forces like simple laboratory dissolver stirrers generate only small mechanical forces while high speed stirrers (up to 25000 rpm) and high pressure homogenizers (up to 10^8^ Pa) enable a pronounced deagglomeration [94]. These methods can only be applied at composite viscosities below 10 Pas. Mixer-kneader and extruders allow for the processing of high viscous polymer melts. In the latter case the resulting shear forces depend also on the configuration of the used extruder screws.

As a measure of the deagglomeration capability of a dispersing method, TEM-images as well as the measurement of the optical transmittance can be used. Böhm and coworkers showed the influence of the dispersing method on the optical transmittance in the NIR-range of highly agglomerated nanosized SiO_2_, ZrO_2_, and Al_2_O_3_, dispersed in a MMA/PMMA based reactive resin after polymerization [95]. They found, that with increasing shear forces the optical damping was significantly reduced, e.g., in case of 5 wt % amorphous SiO_2_ in PMMA from 4 dB/mm (blade stirrer) down to 0.44 dB/mm (high speed stirrer). The use of the high pressure homogenizer enabled a further improvement down to values around 0.26 dB/mm [96]. TEM-images of samples applying either the high speed stirrer or the high pressure homogenizer showed a difference in the agglomeration behavior of the hydrophobic SiO_2_ in PMMA (see Figure 3). A comparable optical damping decay was measured for ZrO_2_ and Al_2_O_3_, dispersed in PMMA [95,96].

After dispersing, a reagglomeration forming micron sized soft agglomerates has to be prevented. A surface hydrophobization using physisorption or chemisorption causes a steric stabilization enabling a repulsive interaction of the particles [12]. The treatment of the hydroxyl-terminated SiO_2_, ZrO_2_ or Al_2_O_3_ with organosilanes yields via chemisorption a hydrophobic surface [12,97]. If the organosilanes carry a reactive, polymerizable functionality, the surface modified particle can be attached to the resulting polymer backbone or network. A short review dealing with these grafting techniques was published by Rong and coworkers in 2006 [98]. Dispersants or surfactants are amphiphilic molecules with a polar and a nonpolar molecular moiety. They are attached physically (physisorption) via van-der-Waals-forces or hydrogen bridges to the particles forming a hydrophobic surface also. A comprehensive overview of the different surfactants and the application possibilities was given by Karsa [99]. The rheological behavior of polymer-nanoparticle composites was in the main research focus in the last 20 years. In an early work Cheng and coworkers [100] described the impact of the particle size distribution of nanosized SiO_2_ (primary particle size 20 nm, agglomerates 50 nm), dispersed in a low viscous methacrylate mixture for dental applications on the rheological behavior. They compared these dispersions with related ones containing coarse and medium sized particles. All systems showed a non-Newtonian flow; the addition of the nanosized SiO_2_ caused a larger viscosity increase than the medium sized at the identical solid loads. Applying the different empirical descriptions for the estimation of the critical filler load, the lowest values were found for nanoparticles [100]. Wetting agents, also named surfactants or dispersants, possess a strong influence on the composite rheology due to a reduced inter-particle friction [101,102]. Song and Evans measured the influence of different dispersants on wax-nanosized ZrO_2_-dispersions. Despite that ZrO_2_ possessed a small average particle size of 70 nm, the specific surface area was relatively low with a numerical value around 22 m^2^/g [103]. For comparison, ZrO_2_ grades with larger average particle sizes and small specific surface areas around 4–7 m^2^/g were considered also. The use of different dispersants, here stearic acid and a commercial product (KD5, ICI Surfactants, UK), showed a pronounced influence on the solid load dependent viscosity of the nanosized ZrO_2_-based composites, in case of the coarse ZrO_2_ the composite viscosity was less affected. At constant load the composites containing the nanofiller showed significant higher viscosities than the composites with the coarse filler. This behavior can be attributed to the large specific surface area of the nanosized filler [103]. With respect to the lithographic (ink-jet) printing of ceramics, nanosized TiO_2_ (average particle size around 200 nm) was dispersed in an acrylic-based ink with the aid of different commercial dispersants (concentration 2.0 mg/m^2^ filler specific surface area) up to a solid load of 79 wt % (45 vol %) [104]. All composites exhibited a pronounced pseudoplastic flow, which required a printing at high shear rates of the ink. The change of the viscosity with load was at low TiO_2_ concentrations moderate and increased disproportionately at concentrations higher than 60 wt % [104].

A comparison of the particle size, particle size distribution and specific surface area of different commercially available micro- and nanosized Al_2_O_3_ on the composite rheology using an unsaturated polyester resin as matrix was published in [105]. Mainly the filler’s specific surface area determined the resulting accessible load and the composite flow behavior. While micron sized Al_2_O_3_ with specific surface areas below 10 m^2^/g allowed composites with a solid load around 40 vol %, nanosized Al_2_O_3_ with a specific surface area of 107 m^2^/g enabled only mixtures with 8 vol % using an unsaturated polyester resin as polymer matrix [105]. The flow activation energy, which is a measure of the temperature influence on the viscosity, showed a pronounced dependency on the nanosized Al_2_O_3_ content and increased with load significantly. The strong impact of the nanosized Al_2_O_3_ on the flow properties can be deduced from the very large specific surface area, the resulting large interfacial layer to the binder and the reduced polymer chain mobility [35]. The influence of the surface polarity on unsaturated polyester-nanosized SiO_2_-composites was characterized by shear viscosity measurements [106]. While the addition of hydrophilic SiO_2_ (primary particle size 12–20 nm) caused a pronounced viscosity increase and small accessible maximum filler load <3 vol %, a hydrophobic SiO_2_ (Aerosil^®^ R8200, primary particle size 12 nm) yielded a moderate viscosity rise up to a solid content of 8 vol %. Quite interesting is the influence on the flow activation energy. With increasing R8200 concentration a pronounced reduction of the flow activation energy can be calculated from temperature dependent viscosity data, which means a reduced temperature influence on the composite viscosity. This phenomenon can be explained by the improved attractive interaction of the hydrophobic filler (specific surface area of 142 m^2^/g) with the hydrophobic polymer matrix and the resulting reduced polymer chain mobility. A similar behavior was observed for a hydrophobic TiO_2_ [107]. Again the large hydrophobic surface area caused an improved attractive particle-matrix interaction.

Summarizing the mentioned literature, the following aspects influence significantly the resulting polymer-nanoparticle composite properties, mainly attributed to the pronounced nanoparticle agglomeration and extreme large specific surface area:

- shear forces during compounding
- particle surface chemistry and polarity
- interaction between bulk polymer and interfacial-polymer layer as well as interaction between interfacial-polymer layer and ceramic nanoparticles.

## 5. Thermomechanical Composite Properties

For many years, micron sized fillers have been used for the reinforcement of the polymers poor mechanical properties. In a rough approximation, the resulting composite properties correlates with the filler volume content in the matrix. In contrast, the use of nanofillers with particle or agglomerate sizes below 100 nm and primary particles below 30 nm does not follow this simple approach in all cases, because due to size effects the following additional aspects have to be considered:

- particle shape, agglomeration, and size distribution
- particle specific surface area and related surface chemistry
- particle-polymer matrix interface and interaction
- compounding method and related shear forces.

Quite often the influence of nanoparticles on the polymer properties is not unique, but in general some trends can be observed. Jordan and coworkers collected in a very comprehensive overview the main trends of the nanofillers impact on the resulting composite mechanical behavior, considering attractive as well as repulsive interactions of the filler with an amorphous or crystalline polymer matrix [108]. Table 3 gives a selection of the influence trend of nanoparticles on the mechanical properties of amorphous polymers taken from [108]. Table 4 lists the related information for semicrystalline polymers, also taken from [108].

**Table 3:** Impact of nanoparticles on composite properties with amorphous polymer matrix.

Item	Polymer-filler interaction	Impact
Elastic modulus	Attractive/repulsive	Increase with volume fraction
	Attractive/repulsive	Increase with size decrease
Density/volume	Attractive	Increased volume as size decreases
	Repulsive	n.a.
Glass transition temperature	Attractive	Increase with size decrease
	Repulsive	Level until 0.5%, drops off level from 1–10%

**Table 4:** Impact of nanoparticles on composite properties with semicrystalline polymer matrix.

Item	Polymer-filler interaction	Impact
Elastic modulus	Attractive/repulsive	Increase with volume fraction
	Attractive/repulsive	Increase with size decrease
Density/volume	Attractive	Increased volume as size decreases
	Repulsive	n.a.
Glass transition temperature	Attractive	Decrease with addition of particles
	Repulsive	n.a.
Crystallinity	Attractive/repulsive	No major effect

In the following, a few examples demonstrate the influence of nanoparticles on the phase behavior, elastic modulus, scratch resistance, hardness and elastic properties. A comprehensive review covering the impact of different nanoparticle types like clay, carbon nanotubes and spherical particles on the mechanical properties of polymer nanocomposites was published by Tjong in 2006 [109].

### 5.1. Glass transition temperature and coefficient of thermal expansion

The Rensselaer group of Siegel investigated the influence of coated and uncoated Al_2_O_3_ (average particle size around 39 nm) on the glass transition (TG) behavior of PMMA [110,111]. They found, that uncoated Al_2_O_3_ caused, at concentrations greater than 0.5 wt %, a significant TG-drop of around 25 °C. The silanization of the Al_2_O_3_ enabled a constant TG even at higher Al_2_O_3_ load of 10 wt %. In contrast, the Hu group found that the addition of hydrophobic nanosized SiO_2_ (Aerosil^®^) to PMMA yielded a pronounced increase of the glass transition temperature up to 15 °C at a solid load of 4 wt % due to a strong attractive interaction of the non-polar nanoparticle with a huge specific surface area up to 200 m^2^/g and the non-polar polymer [112]. The decomposition temperatures of the composites were elevated remarkably even at low SiO_2_ contents. Both groups used sonification in MMA as dispersing method prior to the polymerization to the final PMMA-based composite [110,112]. The addition of two different nanosized Al_2_O_3_ (primary particle sizes 13 and 38 nm, solid load up to 10 wt %) to a methylmethacrylate based reactive resin caused after polymerization only a slight drop of TG [113]. The coefficient of thermal expansion (CTE) was more affected by the 13 nm Al_2_O_3_, which can be explained by the larger specific surface area of 107 m^2^/g than the 38 nm Al_2_O_3_ with only 34 m^2^/g enabling pronounced polymer chain immobility.

Although a TG-increase could not be detected in PMMA/SiO_2_ nanocomposites by thermal analysis (DSC), dielectric and IR-spectroscopy gave evidence for a strong attractive interaction of the nanoparticles with the polymer chains by means of reduced chain movement and longer relaxation times [88]. The direct chemical bonding of monodisperse nanosized SiO_2_ to a polymer backbone, here polystyrene (PS), via grafting, caused a TG increase up to 6 °C at 2 wt %, while the simple physical mixing via sonification generated a slight TG decay [114]. Composites, consisting of nanosized Al_2_O_3_ and the semicrystalline high performance polymer polyetheretherketone (PEEK), fabricated by wet chemical methods followed by hot pressing, showed an elevated decomposition temperature in comparison to the neat polymer [115]. In contrast, the melting temperature was not affected by the addition of the nanosized Al_2_O_3_ even at very high load (30 wt %). The crystallization temperature increased slightly with load. Due to the increasing ceramic content the CTE was reduced [115].

Chen and coworkers [70] found, that small amounts (5 wt %) of the nanofiller induced a higher thermal stability, while larger Al_2_O_3_ concentrations (9 wt %) caused a pronounced weight loss even at low temperatures [70]. Same behavior could be found for TG. A TG increase up to Al_2_O_3_ filler concentrations of 5 wt % were reported by Omrani and coworkers as well [71]. Surface modified nanosized SiO_2_ monospheres with an average diameter of 400 nm, dispersed in an epoxy matrix, caused an increase of TG as well as a reduction of the CTE with solid content [116]. A chemical bonding to the polymer amplified these effects.

Summarizing the data the following statements can be made:

- the addition of ceramic fillers lowers the CTE
- an increase of TG can be observed if an attractive interaction of the nanofiller with the polymer matrix by physic- or chemisorption is given
- a decrease of TG occurs if the nanoparticle has a repulsive interaction with the matrix.

The results are in good agreement with the statements given in section 2.2 (*Polymer-nanoparticle interface*) and the trends quoted by Jordan [108].

### 5.2. Elastic modulus, tensile strength, flexural strength and impact performance

Hot compression molding of surface modified SiO_2_ (primary particle size 12 nm) or Al_2_O_3_ (primary particle sizes 15 and 90 nm), mixed with micron sized PEEK powder, was used for the fabrication of test specimens for mechanical testing [73]. The authors investigated the influence of different mixing methods (direct mechanical mixing, liquid-solid mechanical dispersing, sonification, ball milling) and particle sizes on the composite properties. In agreement to results listed earlier the addition of the nanofillers to PEEK caused an increase of TG but no remarkable change of the melting behavior. The amount of crystalline domains in the polymer decreased. The authors found, that tensile and compressive strength increased with Al_2_O_3_ particle diameter while the flexural strength was not affected. Comparable trends could be detected for the Al_2_O_3_ and SiO_2_ fillers load increase. Both materials showed an optimized concentration of 5 wt % for a raise of the tensile, compressive and impact strength; but a further filler concentration increase yielded a strength reduction. A nanofiller surface modification using different coupling agents did not result in a unique trend of mechanical property reinforcement or weakening. A clear influence of the dispersing method on the investigated mechanical properties was not found [73].

Surface modified nanosized Al_2_O_3_ (primary particle size 39 nm), dispersed in PMMA, caused an increase in strain-to-failure over 28% at a solid load of 5 wt % enabling a ductile flow in the glassy state [117]. Thin films, consisting of up to 22.8 wt % SiO_2_ and polyamide 6, were investigated with respect to their mechanical properties [118]. A significant change of the viscoelastic properties (increase of storage and loss modulus) with increasing load as well as of the Young’s modulus as function of solid load and temperature was reported. The mechanical properties of an *in situ* synthesized and surface modified nanosized TiO_2_ using acrylates as reactive resin matrix were investigated after polymerization [82]. The sol-gel based nanoparticle synthesis and the *in situ* composite formation avoided particle agglomeration guaranteeing a homogenous particle distribution in the matrix. Increasing TiO_2_ content caused an increase of hardness (from 0.030 GPa for the pure polymer and 0.198 GPa for a TiO_2_ content of 10 wt %) and Young’s modulus (from 2.83 GPa for the pure polymer and 4.98 GPa for a TiO_2_ content of 10 wt %). The thermal stability was improved also, an increase of the refractive index in the visible range from 1.5 up to 1.8 (TiO_2_ load 50 wt %) due to the high intrinsic refractive index value of TiO_2_ accompanied with a good optical transmittance was measured [82].

The effect of the nanoparticle size and amount on resulting mechanical composite properties were measured by Cannillo *et al.* [72]. Fracture images showed a strong bond between the surface-modified SiO_2_ and the polymer matrix. Tensile tests showed an improvement of the Young’s modulus with solid load, in case of untreated, hydrophilic SiO_2_ no reinforcement was observed. Hence, for a numerical description of the mechanical behavior using FEM-methods an interfacial layer between the filler and the matrix had to be assumed. Cho and coworkers found, that a pronounced dependence of the mechanical properties like Young’s modulus and tensile strength is given [119]. Surface coated nanosized Fe_2_O_3_ with a nominal particle size of 9 nm, dispersed in an epoxy matrix, induced an improved thermal stability of the resulting composite, a significant TG increase of 15 °C at a 10 wt % solid load as well as an improved pencil hardness [120]. The properties of nanosized SiO_2_-latex-composites are described by Oberdisse [121].

As a short resume the particle size, size distribution, specific surface area, particle load, degree of dispersion, and the particle morphology determine especially the composite’s mechanical behavior [122]. The formation of disordered aggregates and agglomerates generating flexible micron sized clusters instead of isolated nanosized particles affect directly the mechanical properties. Hence a pronounced modulus enhancement in thermoplastic polymers is difficult.

### 5.3. Scratch resistance, wear and creep properties

Ng and coworkers [123] compared the influence of micron-sized and nanosized TiO_2_ (10 wt %, average particle size 0.24 µm and 32 nm, respectively) on the scratch resistance of an epoxy. They found an improvement of the scratch resistance when using the nanosized in comparison to the micron-sized filled polymer and the neat polymer. Same trend was found for the strain to failure behavior; interestingly at other concentrations no impact relative to the pure polymer of the nanosized TiO_2_ was found. The grafting of nanosized SiO_2_ (average particle size 9 nm) with acrylamide and the dispersion in an epoxy matrix via stirring and sonification yielded composites with improved wear properties and reduced friction coefficient relative to the pure polymer and a composite with the untreated SiO_2_ even at low nanofiller content (~2 vol %) [124]. A slight increase of the glass transition temperature due to an attractive filler-matrix interaction and the resulting reduced polymer chain mobility was observed also. Surprisingly no difference between the uncoated and grafted nanosilica was observed. Highly transparent composites, consisting of surface modified SiO_2_ (Aerosil^®^ 600, average primary particle size 40 nm) and Al_2_O_3_ (average primary particle size: 20 nm), dispersed in an acrylate-based polymer, with improved scratch resistance were synthesized by Bauer *et al.* [125,126]. Melt mixing of polyethyleneterephthalate and nanosized Al_2_O_3_ yielded a composite with slightly increased friction coefficient and reduced wear rate at low filler contents up to 2 wt % [127]. In contrast, a further Al_2_O_3_ addition caused an increase of the wear rate to values significantly higher than the pristine polymer. This optimized filler concentration of 2 wt % correlates with a reduction of the polymers crystallinity [127]. Daseri and coworkers published quite recently a comprehensive overview covering all aspects of wear and scratch resistance in polymer-based nanocomposites [128]. Co-extrusion of nanosized TiO_2_ (primary particle size 21 nm) with polyamide 6,6 yielded composites, which were examined with respect to their creep resistance under ambient conditions and at elevated temperature (50 °C) [129]. Test specimens were fabricated by injection molding. Composites containing 1 vol % TiO_2_ possesses a significant improvement of the creep resistance and a reduction of the creep strain in comparison to the unfilled polyamide.

## 6. Functional Properties and Applications of Nanocomposites

Regarding the applications of nanocomposites fundamental knowledge of functional properties is mandatory. Therefore, we first will highlight optical, magnetic, dielectric, and piezoelectric properties of nanocomposites described in literature, together with their application. A focus of this chapter will be the application of nanocomposites in lithium-ion batteries. Finally, further modern applications of nanocomposites in organic solar cells, as photoresists, or in biomedical sciences will be pointed out.

### 6.1. Optical properties

The modification of the refractive index with coeval preservation of the transmittance is one of the challenges for particle/matrix nanocomposites, and therefore reported quite frequently in literature. Most of the research deals with TiO_2_ nanoparticles, embedded in an organic matrix. This is because all TiO_2_ modifications exhibit an inherent high refractive index, as already explained in section 2.1. In some cases, research groups also use semiconducting nanoparticles as ZnS or PbS with inherent high refractive indices. Table 5 summarizes the various and sometimes very differing results. Due to the extreme specific surface area values of nanoparticles with sizes below 10 nm, high nanoparticle contents cannot be expected. Hence, some of the results published in literature and listed in Table 5 are quite questionable. Additionally, the results cannot always be compared among each other, as:

- especially in the case of TiO_2_ not all authors indicate the phase they use
- different units as wt % or vol % are used for the filler
- surface modified nanoparticles as well as pristine nanoparticles are used
- different particle sizes are used
- different processes for the synthesis of the composites are used
- the influence of remaining precursor residuals is unclear.

As shown in formula (1), the composite’s sum refractive index changes linearly with the filler concentration (all concentrations in vol %), starting with the initial value for the pure polymer [130]. In case of small filler concentrations and almost identical density values for the polymer and the filler or dopant, as described in [130], the relation simplifies to formula (2) (concentrations in wt %).

(1) $$n_{composite}=n_{matrix}*c_{\text{​​​}matrix}\;+n_{filler}*c_{filler}$$

(2) $$n_{composite}=n_{matrix}+n_{filler}*c_{filler}$$

For applications in microoptics, the resulting optical transmittance in the visible, e.g., for consumer electronics, and NIR-range, for optical data transmission using the standard telecommunication wavelength at 1310 and 1550 nm, is also of particular interest. At larger sample thickness the transmittance drops significantly due to primary particle agglomeration and prevents the use of the composites in optical devices. Therefore, the published data has to be carefully controlled with respect to the measured sample thickness, a thin layer of some 100 nm thickness can exhibit excellent optical transmittance values in the visible, which drops almost to zero considering technical dimensions of some mm used in the ASTM standard for transmittance measurements applying Lambert-Beers-law. Ritzhaupt-Kleissl *et al.* [94] showed the influence of particle treatment on optical properties: an *ex situ* silanisation of nanosized Al_2_O_3_, dispersion in a MMA resin and subsequent polymerization yielded better optical transmittance values in the NIR than untreated Al_2_O_3_ applying the high speed stirrer as well as the high pressure homogenizer.

Photoluminescence of nanocomposites is another interesting property reported for several classical nanocomposite systems as well as for nanocomposite particles. Excitation and emission of the composite differ significantly from the pure polymer. This was shown for GaN/polymer nanocomposites [80], and for Al_2_O_3_, Y_2_O_3_, ZnO and SnO_2_/Sb_2_O_3_/Sb_2_O_5_ /polymer thin film nanocomposites [66], respectively. In the latter case the spectra broadened, loose vibronic structure and shifted towards higher photon energies (red-shift). The intensity of the effects is influenced by the polymer used. Polyphenylenevinylene (PPV)-based nanocomposites exhibit a stronger influence than poly[2-(6-cyano-6’-methylheptyloxy)-1,4-phenylene] (CN-PPP)-based nanocomposites. A green photoluminescence was observed for CdSe/polybutylacrylate (PBA) nanocomposites [83]. The emission of the composite also differed significantly from the emission of the polymer. For ZnO/vinyl-ester resins photoluminescence spectra also showed a dependence of nanoparticle loading. As expected, the pristine polymer possessed no luminescence. Interestingly already a particle loading of 1 wt % ZnO showed a significant luminescence. With increasing particle concentration an increase in intensity was observed, but no influence on the emission maximum [131]. ZnO/polybutanediolmonoacrylate (PBDMA) nanocomposites showed an increase of the excitation and the emission wavelengths with increasing particle sizes [132]. A similar relationship was observed for ZnO quantum dots dispersed in PMMA [133]. Du *et al.* [134] investigated also the photoluminescence of ZnO nanoparticles in a PMMA matrix. These authors attributed the UV emission located at 334 nm to the quantum size effects of the nanoparticles, and the photoluminescence peak located at 346 nm to the presence of bound excitons in R-(COO)-ZnO complexes. This is the same mechanism as described for luminescent oxide/PMMA nanoparticles [89,135,136].

**Figure 8:**
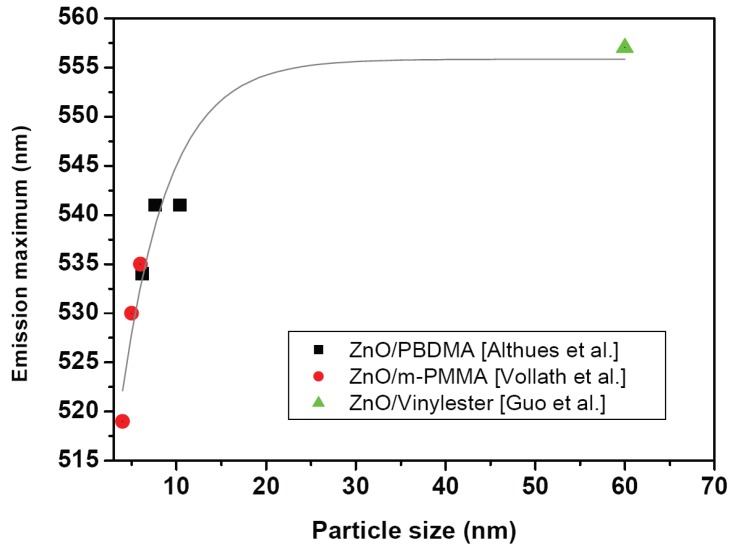
Particle size dependency of the emission maxima of ZnO/PBDMA nanocomposites (Data taken from Althues *et al.* [132]), ZnO/m-PMMA nanocomposite particles (Data taken from Vollath *et al.* [136]) and ZnO/Vinylester nanocomposites (Data taken from Guo *et al.* [131]).

Similar effects are observed at nanocomposite particles with a morphology as described in section 3.2. Photoluminescence was found in TiO_2_/PMMA nanocomposite particles with an emission maximum at 420 nm [137]. A strong luminescence was found in core/shell nanoparticles made of HfO_2_, ZrO_2_, Al_2_O_3_, or ZnO cores and PMMA-shell, respectively [89,136]. The luminescence of nonconducting oxide/polymer nanoparticles was mainly attributed to the presence of carboxylate groups at the interface ceramic/PMMA [135] whereas ZnO as a semiconductor exhibited an inherent luminescence. These authors also found a strong influence of the coating polymer on luminescence. The variation of the coating organic compounds lead to significant changes in the emission spectrum. Furthermore an influence of the particle size on emission maxima and on width of emission lines was found for ZrO_2_ and ZnO. In Figure 8 the size dependency of the emission maxima of photoluminescence for several nanocomposite systems is shown. Data for the three totally different ZnO/Polymer nanocomposite types complement each other. A further development was multifunctional nanocomposite particles where magnetic properties and luminescent properties have been combined in one particle [89]. The particles consisted of a superparamagnetic Fe_2_O_3_ core, coated with an organic dye, and finally with a protective polymer layer. Depending on the organic dye used, photoluminescence could be adjusted.

There is a high application potential for photoluminescent nanocomposites. Examples are epoxy nanocomposites containing ZnO nanoparticles for solid state lightning [138], and ZnO/polymer core/shell nanoparticles for *in vitro* cell imaging [139].

A different approach towards composite with luminescent or lasing properties is the solution or dispersion of rare earth metal complexes in polymer matrices. PMMA, containing a 2.5 wt % Eu-complex was investigated with respect to the 613 nm fluorescence pumped by a 457 nm Ar^+^ laser [140]. Another Eu-complex with particle sizes between 30 and 100 nm was dispersed in a commercial available Araldite GY 251 and the photoluminescence as function of the solid load (up to 5 wt %) was measured at 618 nm [141].

**Table 5:** Modification of refractive indices in particle/matrix nanocomposites.

Nano-Filler	Diameter [nm]	Matrix	Δn, Refractive index increase	Reference
ZnS/PMAA + acetic acid; 50 vol %	ZnS: 3 nm	DMAA/St/DVB	0.023	[78]
ZrO_2_ 50 wt % TiO_2_ 42 wt %	5 nm 7 nm	PC PC	0.067 (at 589 nm) 0.135 (at 589 nm)	[142]
TiO_2_, 60 wt %	Amorphous	Epoxy	0.221	[143]
ME-capped ZnS 30 wt %	ZnS: 3 nm	DMAA/St/DVB	0.048	[87]
TiO_2_ 27.3 vol % TiO_2_ 90 vol %	<10 nm	PS	0.22 0.41	[144]
TiO_2_ acetic acid mod. 10 wt % 30 wt %	~ 15 +/- 10 nm	Epoxy	0.71 (at 633 nm) 0.9 (at 633 nm)	[145]
TiO_2_, 50 wt %	Anatase: 4 nm	Organic silica sol	0.163 (at 633 nm)	[146]
ZrO_2_, 5 wt %	5 – 25 nm	TMP-TGE	0.1 (at 631 nm)	[147]
TiO_2_ surface modified 80 wt % 80 wt %	TiO_2_: 3 – 6 nm	PHE PSTMA	0.23 (at 589 nm) 0.19 (at 633 nm)	[148]
TiO_2_, 65 wt %	amorphous	Epoxy	0.187 (at 633 nm)	[149]
PbS, 41.8 wt %	<10 nm	Polythiourethane	0.481 (at 633 nm)	[150]
Al_2_O_3_-C^®^, 1 wt % ZrO_2_ VP^®^, 0.2 wt %	13 nm 30 nm	PMMA	0.0016 (at 633 nm) 0.0014 (at 633 nm)	[95]
TiO_2_, 35 wt % (=10.5 vol %)	Rutile: 2.5 nm	PVAL	0.088 (at 589 nm)	[151]
Al_2_O_3_-C^®^, 1 wt %	13 nm	High temperature stable PC	0.0043 (at 633 nm) 0.0031 (at 1550 nm	[152]
SiO_2_, 10 wt % Al_2_O_3_-C^®^, 1 wt % Al_2_O_3_, 0.5 wt %	12 nm 13 nm 38 nm	PMMA PMMA PMMA	-0.007 (at 633 nm) 0.007 (at 633 nm) 0.004 (at 633 nm)	[94,96]
ZnO, 7.76 vol %	22 nm	PMMA	0.02 (at 633 nm)	[153]

Abbreviations: PMAA: Polydimethylacrylamide; DMAA: *N,N*-dimethylacrylamide; St: Styrene; DVB: Divinylbenzene; PC: Poly(bisphenol *A* carbonate); ME: 2-mercaptoethanol; TMP-TGE: Trimethylolpropane triglycidyl ether; PHE: Poly(bisphenol-A and epichlorohydrin); PSTMA: copolymer of styrene and maleic anhydride; PVAL: Polyvinylalcohol.

### 6.2. Magnetic properties

Concerning magnetic nanocomposites, two groups of composites can be assessed: those containing metal nanoparticles, and those containing Fe_2_O_3_, Fe_3_O_4_ or ferrite nanoparticles. In most cases the resulting nanocomposites are free of hysteresis, indicating a superparamagnetic material. Kataby *et al.* [154] reported about alcohol-coated amorphous iron nanoparticles without hysteresis. They did not reach saturation, even at an applied magnetic field of 15 T. In contrast, Burke *et al.* [155] found saturation magnetizations ranging from 0.9 to 37.8 Am^2^/kg for different compositions of polymer coated iron nanoparticles. The highest values were associated to the samples with the highest iron loading. Most of their materials exhibited hysteresis at room temperature. Furthermore, they were characterized by a coercivity depending on the core diameter with a maximum at around 20 nm.

Ziolo *et al.* [75,76] measured a saturation magnetization of 15 Am^2^/kg for a polymer nanocomposite containing 21.8 wt % of Fe_2_O_3_. Their material was free of hysteresis at room temperature, and as an additional feature, also optically transparent. Nanocomposites, consisting of magnetic γ-Fe_2_O_3_ nanoparticles in an electroconducting polymer matrix, were free of hysteresis and possessed a saturation magnetization around 58 Am^2^/kg [156]. Biocompatible ferrofluids containing between 10 and 40 wt % Fe_3_O_4_ hybrid nanoparticles were also free from hysteresis [53]. A saturation magnetization around 30 Am^2^/kg and superparamagnetism were found for core/shell Fe_2_O_3_/polymer nanoparticles containing 15.3 wt % of γ-Fe_2_O_3_ [89]. A high saturation magnetization of 69 Am^2^/kg, respectively 54.9 Am^2^/kg was found for two different ε-caprolactone-grafted Fe_3_O_4_ nanocomposites [54]. Nanocomposites containing between 20 and 50 wt % of Fe_2_O_3_ nanoparticles in PPy exhibited saturation magnetizations around 30 - 45 Am^2^/kg. Additionally, an improved electrical conductivity was observed [157], compared to pure PPy. Nanocomposites, containing 12.37 wt % of Fe_3_O_4_ in a polyaniline matrix, were found to be superparamagnetic with a saturation magnetization of 3.88 Am^2^/kg [64]. Optically transparent thin films of block copolymers containing superparamagnetic γ-Fe_2_O_3_ have been developed by Sohn and Cohen [158]. They indicated a saturation magnetization of 0.5 Am^2^/kg for their material containing 2.6 wt % of γ-Fe_2_O_3_.

**Figure 9:**
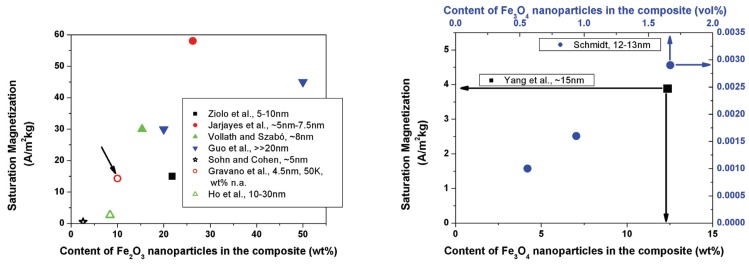
Saturation magnetization of different nanocomposites, containing either Fe_2_O_3_ (left) or Fe_3_O_4_ (right) nanoparticles as magnetic active material, plotted as a function of nanoparticle content. Experimental data are taken from Ziolo *et al.* [75,76], Jarjayes *et al.* [156], Vollath and Szabó [89], Guo *et al.* [157], Sohn and Cohen [158], Gravano *et al.* [55], Ho *et al.* [61], Yang *et al.* [64] and Schmidt [53]. All data are, with exception of those from Gravano *et al.*, acquired at room temperature. This data point (left image) is marked with an arrow, as also composition is unclear. The right image shows two x-axes, one in wt %, and the other in vol %. The y-axes are both saturation magnetization, but with different scales.

Figure 9 summarizes some literature data of different magnetic nanocomposites. As a clear trend, the saturation magnetization is, as expected, a function of the content of magnetic nanoparticles. However, not all authors give straight forward information about the content of magnetic phase. The particle size of the magnetic phase is indicated in the diagram, as far as it is available.

Application potential for superparamagnetic nanocomposites was found as microwave absorbing material [64]. Very recent and advanced developments of organic coated magnetic nanoparticles show application potential mainly in biology, medicine, in biomedicine [54,159,160,161,162], and diagnostics, such as contrast agent for MRI (magnetic resonance imaging) [163,164], in cancer treatment by local hyperthermia [165,166,167], as drug carriers [168,169,170] or as biocompatible ferromagnetic fluid [53,171].

### 6.3. Microelectronic devices

The addition of conductive nanoparticles to polymers has a strong impact on the resulting composite dielectric properties. With respect to the aspired integration of passive electronic devices, like resistors, capacitors and others, into the printed circuit board (PCB), new composite materials have to be developed to meet the following requirements [172,173,174]:

- huge functionality like large capacitance values in case of integrated capacitors
- process compatibility to industrial PCB-fabrication
- abandonment of lead-containing materials
- low overall costs
- high reliability and extended life cycle.

In case of embedded capacitors, suitable materials with high permittivity are needed, achieving, in combination with a further reduction of the capacitor thickness from 100 down to 10 µm, large capacitance values in the range of some nF/mm^2^ [175]. Polymer based-ceramic nanocomposites fulfill the mentioned requirements, if both components possess a high permittivity. Common polymers exhibit only low relative dielectric constant values between 2 and 4 [173]. Epoxies typically used in PCBs shows permittivity values around 3.5 [176]. Ferroelectric polymers like polyvinylidenefluoride (PVDF)-derivatives deliver high-k-values around 60 under ambient conditions, but these polymers cannot be used applying the standard board manufacturing technology [177].

A very promising way to raise the composites dielectric constant is the addition of suitable ferroelectric ceramics with extreme permittivity values like BaTiO_3_ to polymers [173,178]. As the size effects for dielectric properties are in another size regime as the size effects of magnetic or optical properties (see section 2.1), size effects in the nanocomposites are also expected in another size regime. These perowskite-type ceramics show a pronounced dependence of the permittivity from the crystallite size, decreasing grain sizes down to 1 µm yielded a significant increase of the relative dielectric constant in the relevant temperature range between -50 °C and 100 °C [179]. A further size reduction towards the nanoscale caused a lowering of the permittivity again [180]. It can be expected that an optimized grain size can be found between 100 nm and 1 µm. First attempts to find a grain size with improved dielectric properties were published 2008 applying a systematic thermal treatment of nanosized BaTiO_3_ with an average particle size of 85–128 nm [181,182]. The thermal treatment at 1000 °C allowed for a grain size growth and an increase of the dielectric constant from 9 to 25 in a polyester-based composite with a solid load of 60 wt %. The change of the dielectric constant with load in a polyester matrix of different nanosized ceramics like TiO_2_, SnO_2_, ZnO, BaTiO_3_, SrTiO_3_ and others, were published quite recently [172]. In all cases poor permittivity values could be measured. Hence, the thermal treatment was extended to nanosized (69–104 nm) SrTiO_3_, but only a small improvement was measured due to the cubic phase of the ceramic. In contrast the dielectric loss could be significantly reduced by a factor of 3 [183]. Commercially available BaTiO_3_ with different particle size distribution from 100 up to 700 nm (Inframat Advanced Materials) were investigated with respect to their dielectric properties [184]. Composites containing 65 wt % of a 200 nm BaTiO_3_ showed a permittivity around 15.

As a short resume, high-k-ceramics, like BaTiO_3_ and others, possess at the nanoscale a non-equilibrium crystal lattice with reduced permittivity values. A controlled grain size growth by thermal treatment towards the microscale helps finding an optimized grain size enabling higher permittivity values [182].

### 6.4. Piezoelectric actuators and sensors

Piezoelectric ceramics like lead zirconate titanate (PZT) are widely used in microsystems technologies. They are characterized by outstanding applicability as microactuator converting electrical energy into mechanical movement, vice versa as sensor or for energy harvesting as well as for exploiting the pyroelectric properties [178,185,186]. Despite the simplified device realization in comparison to pure ceramic samples, polymer-PZT-composites are seldom investigated. To achieve an effective mechanical response after applying an electrical field the device must be electrically conductive at the surface when connected to a frequency generator. In addition the solid load should exceed the percolation threshold; otherwise the mechanical response of the PZT domains would be mainly absorbed by the polymer matrix. PVDF is the only commercially available polymer showing piezoelectricity. Bloss and coworkers investigated composites, consisting of polyvinylidendifluoride-trifluorethylene (PVDF-TrFE) and PZT, prepared by solvent casting and subsequent compression molding, with respect to their piezoelectric and pyroelectric behavior [187]. The resulting composite pyroelectric and piezoelectric coefficients cannot be derived from a simple mixing rule applying the individual material values of the matrix and the active filler. The dielectric constant as well as the mechanical properties influenced the composite properties, also [187]. In addition, theoretical calculations delivered the impact of the piezoelectric anisotropy of the active filler on the resulting composite behavior [188].

### 6.5. Lithium-ion batteries

In the last years the development of electrical storage systems like primary and secondary batteries has gained a worldwide significance for portable electronic devices as well as for hybrid or full-electric driven vehicles [189]. Especially the future development of large-scale lithium-ion-batteries towards a powerful and safe storage system with huge energy density and specific power will enable a sustainable individual mobility [190]. With respect to application as rechargeable battery (secondary battery) the following criteria have to be fulfilled [191,192]:

- mechanical and chemical stability of the used electrode and electrolyte materials
- huge energy storage capability
- wide temperature range of operation (-40–85 °C)
- negligible self-discharge
- flat shape of the discharge curve
- short charge time
- long cycle life time with almost unchanged capacity
- low costs
- enhanced safety especially inflammability.

Following the current technical setup of lithium-ion-batteries the basic functional elements are:

- anode: pure lithium metal or more common graphite
- cathode: spinel-type lithium-metal oxides like LiCoO_2_ or LiMn_2_O_4_
- electrolyte: highly polar, aprotic low-viscous organic solvents mixtures containing a conducting salt like LiClO_4_, LiPF_6_ or LiBF_4_
- separator: physical barrier between the electrodes avoiding short-circuit and supporting a mechanical stability, consisting of a porous inert material filled with e.g., a polymer-gel.

Besides the mentioned low viscous, organic solvent based electrolytes, which suffer from pronounced flammability, which is inacceptable in automotive usage, polymer-gels, solid polymers as well as polymer composites can be found as electrolytes in lithium-ion-batteries; mostly in small-sized devices like mobile phones or in niche applications as in model airplanes. These electrolytes possess an enhanced safety due to an improved protection against leakage, no internal shorting, reduced combustibility and better freedom of design [192]. The basic requirements for the use of polymer-based electrolytes in modern lithium-ion-batteries are a high ionic conductivity in a wide temperature range, an acceptable mechanical stability, a huge lithium ion transference number, improved thermal stability in the aspired operation range, formation of a porous solid-electrolyte-interface (SEI), which is permeable for lithium ions and guarantees a reproducible charging-discharging cycling behavior and finally a good wetting of the electrodes [193,194]. Three different types of polymer-based electrolytes have been developed: solvent-free polymer electrolytes, gel polymer electrolytes and polymer composite electrolytes. In the latter case ceramic nanoparticles, dispersed in the polymer matrix, have been widely under investigation for their use as additives enabling an ionic conductivity increase especially under ambient conditions [194,195].

In the following the impact of nanosized ceramics on the development of lithium-ion-batteries will be discussed in more detail.

#### 6.5.1. Polymer-nanocomposite electrolytes applying passive ceramic nanofillers

In the early seventies of the last century the first solid solvent-free polymer electrolyte was presented in a short note by Fenton and coworkers [196]. To achieve an acceptable ionic conductivity suitable alkali salts must be soluble in the polymer. The polar molecular structure of polyethyleneoxide (PEO) enables the formation of stable polymer-salt-complexes PEO-LiX. Pure PEO possesses, under ambient conditions, a poor ionic conductivity of 10^−8^ S/cm, which can be improved by the addition of lithium halides by a factor of 100 [193,194]. The semicrystalline PEO showed, depending on the average molecular weight, a TG around -64 °C and a melting temperature around 65 °C [193]. In the intermediate range the presence of semicrystalline domains hindered the lithium-ion mobility resulting in a poor ionic conductivity. The addition of organic plasticizers like highly polar solvents (ethylenecarbonate, propylenecarbonate, dimethylcarbonate, and others) with a relative permittivity around 50–70 for better salt solvation or ceramic particles suppressed the crystallization enabling an enhanced amount of amorphous domains [197]. These early research efforts showed that the application of submicron sized ceramics instead of micron-sized fillers forming polymer-nanoparticle composites is favorable enabling an ionic conductivity around 10^−5^ S/cm near room temperature [198,199]. The latter authors found, that the addition of Al_2_O_3_ yielded an increase of the ionic conductivity only below the PEOs melting temperature. Passing the phase transition the filler causes a stiffening of the polymer host, and the reduced polymer chain mobility lowered the ionic conductivity [199]. Przyluski and coworkers used the effective medium theory for a description of the temperature, composition, grain size and solid filler load of the composite electrolyte conductivity [200]. The model predicted the influence of the grain size and the filler concentration on the conductivity. The conductivity increase could be addressed to the formation of a flexible amorphous layer at the interface between the polymer matrix and the fine particles [200].

A direct comparison of nanosized (13 nm) and micron-sized (<10 µm) Al_2_O_3_ as passive fillers, dispersed in PEO-LiBF_4_, was investigated by Krawiec *et al.* [201]. As observed earlier, an optimized nanofiller content (around 10 wt %) for a pronounced ambient temperature ionic conductivity increase was found. The addition of nanosized TiO_2_ and Al_2_O_3_ enabled a ionic conductivity of 10^−5^ S/cm at 30 °C and of 10^−4^ S/cm at 50 °C [202]. The ionic conductivity was at all investigated temperatures (below and beyond the melting temperature around 60 °C) higher than the electrolyte without ceramic filler. The large surface area of the applied nanofillers did not affect the ionic conductivity remarkably in a negative way and yielded an improved mechanical stability of the electrolyte applied as a polymer membrane usable as separator [202]. Best and coworkers researched the impact of nanosized TiO_2_ (P25, Degussa, today Evonik Industries) on two different amorphous polyethyleneglycol (PEG)-LiX mixtures (X: LiClO_4_, Li[(CF_3_SO_2_)_2_N] prior and after polymerization to the final polymer [203]. In case of high conducting salt concentrations the addition of the P25 increased the ionic conductivity after polymerization. The authors explained the increase by the influence of the nanofiller on the ion-aggregation reducing the ion-pair formation. A comprehensive investigation of the influence of 30 micro- and nanofillers (Al_2_O_3_, AlN, SiO_2_, TiO_2_, ZrO_2_, and others) on the ionic conductivity, TG and melting behavior of PEO-LiClO_4_ complexes was published by Kim *et al.* [204]. Unfortunately, only a correlation between TG and melting temperature could be verified. Croce and coworkers measured the impact of passive (TiO_2_, Al_2_O_3_) and active fillers (LiAlO_2_) on the conductivity and the lithium interface resistance [205]. In both cases the passive nanofillers delivered better values due to their small particle size and large surface area in comparison to the micron-sized active filler. Similar results were described by Appetecchi *et al.* [206]. A solvent-free preparation of the composite electrolyte using hot-pressing as a membrane shaping method and nanosized fumed SiO_2_ or alumina as plasticizers were developed, enabling a simpler cell preparation [207,208].

The longtime research on PEO-based systems showed, that due to the semicrystalline properties a good ionic conductivity under ambient conditions cannot be achieved. Hence, alternative polymer systems, polymer blends and composites have been investigated for the last years. Wachtler and coworkers developed polymer gels consisting of PVDF as matrix, the polar organic solvents ethylenecarbonate and propylenecarbonate as plasticizers and nanosized hydrophilic fumed SiO_2_ (7 nm) as mechanical stabilizer [209]. They found, that the addition of the nanosized filler did not affect the electrochemical properties, but a strong influence of the mechanical strength as function of the SiO_2_ load could be detected. A polymer gel, consisting of a poly(acrylnitril-methacrylate) copolymer and LiClO_4_, solved in an ethylenecarbonate/propylenecarbonate-mixture, was placed as electrolyte on a polyethylene separator in a cell with a lithium metal anode [210]. AlI_3_ was added for the suppression of dendritic deposition of lithium during cycling. In addition to the enhanced cycling behavior the authors measured a good ionic conductivity under ambient conditions of 7.6 × 10^−4^ S/cm and an electrochemical stability window around 5 V [210]. A comprehensive review on separators used in electrolytes for lithium-ion batteries is given in [211].

A completely different electrolyte system used the amorphous copolymer poly(vinylidenefluoride-hexafluoropropylene) (PVDF-HFP), a room temperature ionic liquid (RTIL) and nanosized TiO_2_ [212]. Ionic liquids consist of an organic cation, combined with an inorganic anion like Cl^−^ or large, asymmetric organic anions like [(CF_3_SO_2_)_2_N]^−^, forming stable, low viscous salts under ambient conditions with a negligible vapor pressure and flammability as well as high thermal and electrochemical stability [192,213]. Suitable ionic liquids show an intrinsic ionic conductivity around 10^−3^-10^−4^ S/cm. The authors found, that at concentration of 18 wt % of the nanosized TiO_2_ a maximum ionic conductance of 3.1 × 10^−3^ S/cm could be achieved [212]. A further concentration increase caused a decay of the conductivity due to a stiffening effect, which improves the mechanical stability.

PEG based gel electrolytes, containing either hydrophilic or hydrophobic Aerosils^®^ (A200 and R805 from Evonik) and Li[(CF_3_SO_2_)_2_N] as conducting salt, were been investigated with respect to the corrosion behavior of the electrolyte to the aluminum foils, which are used as current collectors in lithium-ion-batteries [214]. It was demonstrated earlier, that the applied lithium salt caused pronounced aluminum corrosion [215]. The authors found, that the addition of fumed silica hindered aluminum corrosion, especially in case of the hydrophilic Aerosil^®^ A200. In addition of aluminum surface coating, which protects the metal, a moisture scavenging behavior at the hydrophilic surface explained the corrosion protecting effect [214]. Bifunctional (hydrophilic/hydrohobic) surface modified SiO_2_, added to a gel consisting of (PVDF-HFP)-LiPF_6_ in ethylenecarbonate/diethylcarbonate, caused an improved electrochemical electrolyte stability up to 5 V, which can be attributed to electrode surface protection and impurity scavenging [216]. Acrylate-based gel homopolymer electrolytes have been hardly investigated. Krejza and coworkers generated a MMA-LiClO_4_-Al_2_O_3_-nanocomposite by *in situ* polymerization in propylenecarbonate [217]. After polymerization an ionic conductivity value around 3.7 × 10^−4^ S/cm have been measured in the gel.

As a resume passive nanosized fillers are mainly used for the suppression of crystallization in case of PEO-based electrolytes enabling a higher ionic conductivity under ambient conditions or for an improvement of the mechanical stability of polymer gels. The impact of nanofillers like clay, carbon nanotubes and spherical particles on the crystallization properties of different polymers like PVDF, nylon, PS, PEO, and others, are reviewed in 2006 [218]. The addition of nanosized ceramics to amorphous polymers or polymer gels does not affect significantly the ionic conductivity, but a better cycling stability by forming a protection layer at the electrode’s surface has been observed.

#### 6.5.2. Polymer-nanocomposite electrolytes applying active ceramic nanofillers

At the beginning of the nineties of the last century active micro- and nanosized fillers have been introduced to polymer electrolytes. Active fillers are either ceramic materials, which contribute directly to the lithium ion conductivity - in addition to the conducting salt - by e.g., adding lithium salts like LiAlO_2_, or ceramics with large permittivity for better solvation of the conducting salt. Capuano and coworkers added fine LiAlO_2_ to a PEO-LiClO_4_-complex [219]. The authors found, that an optimized filler concentration, which is lower as the maximum accessible filler load, allowed for the aspired crystallization suppression and an ionic conductivity increase (60 °C: 10^−4^ S/cm). Borghini and coworkers observed also a pronounced suppression of the polymer crystallization rate and a better ambient temperature ionic conductivity in the system PEO-Li[(CF_3_SO_2_)_2_N] using submicron γ-LiAlO_2_ [220]. The same filler was used for investigations on the two different polymer-conducting salt complexes PEO-LiCF_3_SO_3_ and PEO-LiBF_4_ [221,222]. An improved cycling efficiency around 99% was observed, also a good stability against the lithium metal anode even at elevated temperatures around 90 °C [221,222,223,224]. A more complex polymer blend, consisting of polyethyleneglycol-diacrylate/PVDF/PMMA, a mixture of LiPF_6_/LiCF_3_SO_3_ and LiAlO_2_ or BaTiO_3_ as nanosized fillers, showed an improved ionic conductivity, interfacial stability as well as better cycling performance [225]. Sutto and coworkers investigated the influence of micro- and nanosized BaTiO_3_ in an electrolyte consisting of the amorphous copolymer PVDF-HFP and a room temperature ionic liquid [212]. They found a pronounced increase of the ionic conductivity with decreasing particle size from 1 µm down to 60 nm by a factor of almost 100. Quite recently, a high ionic conductivity of 8.1 × 10^−3^ S/cm using a polymer composite (PVDF-HFP/LiClO_4_) with 50 nm LiAlO_2_ could be measured by Sundaram and Subramania [226].

In summary the addition of active micro- and nanosized fillers, mainly LiAlO_2_, causes in case of PEO-based electrolytes an improvement of the ionic conductivity by the suppression of the PEO’s crystallization below 60 °C. An enhancement of the cycling behavior due to an improved interfacial stability was also observed. Gels on the basis of PVDF-HFP copolymers show higher ionic conductivity due to nanosized ceramic filler also. The addition of micron sized filler causes negligible effects. This can be explained by the large specific surface area of the nanofillers enabling longer conducting paths for the lithium-ions.

#### 6.5.3. Nanocomposites as electrodes and supercapacitors

It was already shown in Table 2 that nanocomposites based on inorganic nanoparticles and carbon became of particular interest as anode materials in lithium-ion-batteries. Nanoscaled materials are expected to enhance the performance significantly as the reduced dimensions increase the rate of lithium insertion and removal due to the extremely short diffusion lengths [227]. Additionally, electron transport within the particles is enhanced, and the high surface areas permit high contact areas with the electrolytes. Up to now, graphite is the standard material for anodes in commercial lithium-ion-batteries with a theoretical capacity of 372 mAh/g and a more or less stable cycling stability. Nanomaterials based on tin dioxide (SnO_2_) possess very promising potential as anode material because they exhibit in principle much higher specific capacities (790 mAh/g). Unfortunately, bulk and pristine SnO_2_ anode material shows very poor long-term cycle stability due to internal stresses caused by the large volume change (>200%) during the alloying process from Li and Sn forming Li_4.4_Sn. This alloying process results in cracks and loss of electrical contact at the anode. Many research efforts therefore focus on nanocomposite materials based on SnO_2_ or TiO_2_, combined with carbon, graphite, or graphene, acting as:

- a barrier to suppress the aggregation of active particles
- a buffering matrix to relax the volume expansion during the lithiation/delithiation process
- an improvement of the conductance of the electronic material.

The following explanations focus on anodes, fabricated from nanocomposite powders using additionally carbon black for conductivity and a binder. Wang *et al.* showed [228] that the cycling stability can be improved significantly compared to pristine SnO_2_ when using 7 to 10 nm SnO_2_ nanoparticles dispersed in graphite. They also could enhance the specific capacity of these nanocomposites compared to graphite. Due to further particle size reduction of SnO_2_ in the range of 2 to 3 nm by applying a microwave-assisted synthesis, the cycling stability could further be enhanced [229]. Nevertheless, after a certain amount of cycles the capacity decreased below the one of graphite. Fu *et al.* [56] developed TiO_2_/C core/shell nanocomposites using commercial TiO_2_ nanoparticles. Conductive additive and additional binder is needed with this setup. Their material also showed enhanced cycling stability compared to the pure metal oxide. Also nanocomposites using PPy show an enhanced cycling stability compared to SnO_2_. This was shown by Yuan *et al.* [230]. Recently, Qiao *et al.* [59] could realize anodes of SnO_2_ at C core/shell spheres as active material, retaining their cycle stability after 18 cycles, and a specific capacity still higher than carbon. The authors attributed this stability to the presence of the carbon shell, enhancing the conductivity of SnO_2_ cores and suppressing the aggregation of active particles during cycling. A very promising development is metallic Sn nanoparticles encapsulated in an elastic hollow carbon sphere [65]. With this type of material, even after the 100th cycle 66.2 % of the theoretical specific capacity was retained, with a capacity significantly higher than the one of graphite. Carbon encapsulation of SnO_2_ [58] also results in higher reversible specific capacity, and a capacity of over 400 mAh/g after 30 cycles. Liu *et al.* [231] report about the development of SnO_2_/C nanocomposite anodes with superior cycling capacity. They could realize an anode material containing 75 wt % of SnO_2_ nanoparticles with sizes around 3–4 nm, highly dispersed in a carbon precursor matrix derived from glucose. This material was characterized by a stable relative charge capacity of 610 mAh/g even after 200 cycles.

Quite recently another interesting approach is presented by Yao *et al.* [232] developing an anode material based on SnO_2_ nanoparticles on a graphene matrix, also exhibiting an improved cycling stability compared to bare SnO_2_ nanoparticles. Table 6 summarizes the characteristic features of the newly developed nanocomposite anode materials.

A further development in the field of lithium-ion battery is the application of nanocomposites as supercapacitors. Recently, Li *et al.* [233] fabricated conductive graphene/SnO_2_ nanocomposites exhibiting enhanced and stable specific capacitance compared to graphene. Hu *et al.* [234] prepared polyaniline/SnO_2_ nanocomposites, with SnO_2_ nanoparticles embedded in a netlike polyaniline network. This composite material is characterized by a 3 times higher energy storage density compared to pure SnO_2_ and a specific capacitance decay of only 4.5 % after 500 cycles.

### 6.6. Organic solar cells and intrinsic conductive polymer nanocomposites

Organic solar cells are widely under investigation since the early publication by O’Regan and Graetzel in 1991 dealing with the photocurrent generation after photon absorption by an organic dye and electron injection into the conduction band of a n-type semiconductor like TiO_2_ [235]. Modern dye-sensitized organic solar cell uses polymer electrolytes quite similar to systems used in lithium-ion-batteries [236]. Quite recently the positive influence of nanosized TiO_2_ (13 nm, Degussa/Evonik), dispersed in PVDF, on the long term stability of the solar cell was demonstrated [237].

Intrinsic conductive polymers have gained more importance at the latest since the Noble prize for Alan J. Heeger in chemistry in 2000. These polymers, like polyaniline (PANI), polythiophene (PTP), PPy or PPV, are expected to be used in organic solar cells, display technology, photodiodes or batteries. In the last years the addition of nanosized ceramics like SiO_2_, Al_2_O_3_, or TiO_2_ for a chemical stabilization, an improvement of the physical properties as well as a better dispersibility in water or organic solvents has been investigated. Ballav and Biswas prepared composites consisting of PTP and nanosized alumina (particle size: 22–74 nm) [238]. They found a slight increase in the electrical conductivity and an improved thermal stability in contrast to the pure homopolymer. In contrast to the neat polymer, the composite showed the formation of stable suspensions in water and isopropanol. Yang and coworkers investigated the influence of nanosized SiO_2_ (20 nm) and TiO_2_ (20 nm) on the stability of the PPV-precursor molecule [239]. The authors claimed that the addition of the nanoceramics can improve the stability against photodegradation. Both nanoparticles caused an increase of the conjugation length. The optical and electrical properties of the same composites were measured by Yang *et al.* [240]. They found a pronounced impact of the nanoparticles, their particle size and concentration on the photoluminescence and current density-applied field characteristics. In case of composites consisting of PANI, doped with *in situ* generated nanosized TiO_2_ (mean particle size: 20 nm) up to a solid load of 80 wt %, the electrical conductivity was significantly influenced by the filler [241]. Whereas small amounts (5 wt %) caused a pronounced conductivity increase from 0.016 × 10^3^ S/m for the neat polymer up to a value of 0.704 × 10^3^ S/m, a further TiO_2_ increase yielded a conductivity drop close to the initial value. The large value at low solid load may be attributed to the formation of a network with improved charge transport.

In solar cells nanoparticles face very different challenges. Hence, requirements are very different, too. The impact of different ceramic nanoparticles on physical properties including conductivity is given in a comprehensive early review [242].

### 6.7. Polymer-nanocomposite-photoresists

In microelectronics and microsystems technology lithography, using suitable photosensitive polymer based resists is one of the most important techniques for the realization of nano- and microstructured layers [185,186]. Two principal reaction mechanisms determine the selection of suitable polymer resists. In case of a positive-tone photoresist, the irradiation of a polymer film through a mask causes a photodegradation of the irradiated areas and a pronounced solubility of the material. The unexposed resist remains insoluble, a positive image of the mask structure appear after solvent development. In case of a negative-tone photoresist, the uncured resist polymerizes under irradiation forming a negative image of the mask structure. Typical representatives are PMMA for a positive resist and epoxides (e.g., SU8, Microchem. Corp.) for a negative resist.

The addition of nanosized ceramic fillers to a photoresist is driven by the following aspired aspects:

- improved sensitivity to electromagnetic radiation of a certain wavelength region
- improved resolution
- improved chemical resist stability
- improved mechanical stability during processing
- tailoring of the coefficient of thermal expansion
- introduction of new functionalities like electrical conductivity or magnetic properties
- direct fabrication of microstructured ceramic or metal components via microstereo-lithography (rapid prototyping).

As an example, the addition of nanosized SiO_2_ improved significantly the accessible resolution of standard resists for electron beam lithography from 131 nm down to 47 nm [243]. Surface modified nanosized SiO_2_ (10–50 nm particle size) helped to improve the process stability of a photosensitive fluorinated polyimide by a TG and decomposition temperature increase by reduction of the polymer chain mobility due to the enhanced interaction of the particle large surface area and the polymer matrix [244]. Furthermore, the coefficient of thermal expansion was reduced by the filler. The new composite allowed for a direct fabrication of high temperature stable optical devices by UV-lithography. The addition of alumina with an average particle size of 400 nm to SU8 up to a solid load of 42 vol % enabled the direct fabrication of high aspect ratio ceramic microcomponents by deep x-ray lithography and thermal post-processing [245]. The same resist, modified with highly agglomerated nanosized silver particles (load up to 40 vol %), was used for the development of an electrically conductive resist, which can be patterned by the UV-lithography process [246]. The percolation threshold for conductivity was measured to be around a low silver content around 6 vol %. For further reading a comprehensive overview about polymer-filled composites applied as resist systems can be found in [247].

**Table 6:** Overview on performance of new nanocomposite anode materials for Li-ion battery.

Material	Initial specific capacity [mAh/g]	# cycles/capacity retention	# cycles/capacity retention	# cycles/capacity retention	Ref.
Pure graphite (C)	300	10/100 %	30/99.1 %	50/97.8 %	[228]
Bulk SnO_2_	652	10/63 %	30/49.8 %	50/31.7 %	[228]
4 wt % SnO_2_ in C	342	10/99.6 %	30/96.6 %	50/88 %	[228]
9.8 wt % SnO_2_ in C	384	10/99.4 %	30/96.3 %	50/88.3 %	[228]
16.5 wt % SnO_2_ in C	428	10/99.2 %	30/90.1 %	50/72.4 %	[228]
14.2 wt % SnO_2_ in C	465	40/90 %	60/80 %		[229]
14.9 wt % SnO_2_ in C	472	40/89 %	60/75 %		[229]
14.4 wt % SnO_2_ in C	460	40/74 %	60/56 %		[229]
TiO_2_	n.a.	10/67.5 %			[56]
TiO_2_/C (87/13)	122	10/96.7 %			[56]
SnO_2_/PPy (81.75/18.25)	562	20/70 %			[230]
SnO_2_	570	20/40 %			[230]
SnO_2_/graphite	633	30/57 %			[57]
SnO_2_ at C	667	18/55 %	30/55 %	40/55 %	[59]
TNHCs	831	10/>96 %	>100/66.2 %		[65]
SnO_2_/C (75/25)	993	50/62 %	200/62 %		[231]
SnO_2_/graphene (40/60)	765	100/66.9 %			[232]

Abbreviations: TNHCs: Tin nanoparticles encapsulated elastic hollow carbon spheres.

### 6.8. Biomedical sciences

New materials with potential biomedical applications have been developed with respect to direct, supplement or substitute the functions of living tissues [248]. Biocompatible materials must fulfill a chemical, biological, and physical as well as a structural, *i.e.,* mechanical behavior, compatibility with the surrounding host tissues. In case of mechanical properties especially the elastic modulus (Young’s modulus), strength, stiffness and optimal load transmission are of particular interest. In addition to polymers, ceramics and metals polymer-composites allow for a tailoring of the aspired physical properties. In the following a few examples for the application of polymer-filler-composites are summarized [248]:

- bone fracture repair: Epoxide-carbon fibers-composite for external fixators
- bone plates and screws: Epoxide, PMMA, polypropylene, polyethylene, PS, Nylon, polybutylterephthalate, PEEK, reinforced with carbon fibers
- joints replacement: Ultrahigh molecular weight polyethylene or PEEK-carbon fibers composites for total hip replacement
- bone cement: PMMA-glass powder
- dental applications: Acrylates, filled with surface modified nanosized SiO_2_ or ZrO_2_
- catheters: Urethanes or silicone rubber, reinforced with nanosized SiO_2_
- prosthetic limbs: Thermosets, reinforced with glass, carbon, or Kevlar fibers.

A comprehensive overview was published by Ramakrishna and coauthors in 2001 [248]. In a recent publication the impact of the particle size, distribution, and geometrical shape on the resulting mechanical properties of different composites suitable as dental filling materials for substituting amalgams was summarized [249]. The authors found, that especially the homogenous nanoparticle distribution in the matrix ensured a long dental filler lifetime. In contrast to composites with passive fillers improving mainly the thermomechanical properties, the ferroelectric PVDF, filled with nanosized (20–60 nm) hydroxyapatite for improved biocompatibility and nanosized (100 nm) BaTiO_3_ with its high dielectric constant, could be used as bioelectroactive bone regeneration composite [250]. PMMA/SiO_2_ nanocomposites containing calcium salts can be used as bioactive bone substitute or as filler for PMMA bone cement [251]. Fluorescent, stable aqueous ZnOat Polymer core/shell nanoparticles exhibit a high potential for application as fluorescent probes *in vitro*, as they are almost nontoxic for human cells [139]. Finally, as already shown in section 6.2., there is a huge application potential for superparamagnetic nanocomposites in the field of biology, medicine, diagnostics and therapy [53,54,159,160,161,162,163,164,165,166,167,168,169,170,171].

## 7. Summary

In classical nanocomposites, the addition of nanofillers to a polymer matrix allows in principle the tailoring of physical properties. The resulting thermal, mechanical, optical, magnetic or conducting properties of the nanocomposites are influenced by the filler properties as well as from the fillers surface properties.

When designing new polymer-nanoparticle composites the following aspects should be considered:

- Size dependent physical properties of the nanoparticles used
- Particle agglomeration
- Maximum accessible shear forces during compounding affects composite properties
- Reproducibility and comparability of composite formation techniques
- Influence of additives like surfactants, plasticizers, and others, on the composite properties
- Beyond target property: side effects on the flow behavior, thermal stability, and others
- Low cost device fabrication by e.g., suitable shaping methods possible?

With respect to the composite properties, and in contrast to micron-sized fillers, the interface between the nanoparticle and the polymer matrix plays a dominant role due the large filler specific surface area. Quite often a nanofiller surface hydrophobization using chemi- or physisorption helps for composite property tailoring. The interesting nanoparticles are in most cases SiO_2_, Al_2_O_3_, ZrO_2_, TiO_2_, Fe_2_O_3_, SnO_2_, or ZnO with high application potential in different fields. In many cases particles improve several composite properties. Due to the huge surface area of the filler and its interfacial polymer layer, the maximum filling content is limited. As there is a complex interplay between matrix, interface and filler a targeted physical property design of new materials can only be realized, if the interface between the nanofiller and the polymer matrix can be controlled on a molecular level very precisely. Then many new potential applications can arise. Other, interesting composite concepts, using core/shell hybrid nanoparticles or microsphere composite nanoparticles, also open a variety of application potential.
